# Enhanced functional connectome of cerebellum in chronic insomnia patients

**DOI:** 10.1002/brb3.3103

**Published:** 2023-06-05

**Authors:** Shiqi Lin, Xi Ye, Yuping Yang, Jingyi Yang, Guang Xu, Xinzhi Wang, Xiaofen Ma

**Affiliations:** ^1^ Department of Radiology Guangdong Second Provincial General Hospital Guangzhou China; ^2^ Institute for Brain Research and Rehabilitation South China Normal University Guangzhou China; ^3^ Department of Neurology Guangdong Second Provincial General Hospital Guangzhou China

**Keywords:** cerebellum, chronic insomnia, functional connectome, graph theory, resting‐state fMRI

## Abstract

**Background:**

Functional abnormalities of the cerebellum have been found to be closely associated with chronic insomnia (CI). However, whether there are abnormalities in the topology of the functional connectome of the cerebellum in these patients is still unknown. This study aimed to investigate topological abnormalities of the cerebellar functional connectome in individuals with CI.

**Materials and Methods:**

We used resting‐state functional magnetic resonance imaging (rs‐fMRI) and graph‐theoretic analysis to construct a functional connectivity matrix and extract topological property features of the cerebellar functional connectome in patients with CI. We examined global and nodal topological property changes in the cerebellar functional connectome in 102 patients with CI (CI group) and 101 healthy participants without insomnia symptoms (HC group) to determine the differences between groups. Correlations between the topological properties of the cerebellar functional connectome and clinical assessments were calculated to confirm the differences between groups.

**Results:**

The cerebellar functional connectome of both CI and HC patients exhibited small‐world properties. The CI group showed higher standardized clustering coefficients at the global properties and higher betweenness centrality in the cerebellar Crus II vermis region at the nodal properties compared with participants in the HC group. However, the topological properties of cerebellar functional connectome abnormalities in the CI group were not significantly different from those in clinical assessments.

**Conclusion:**

Our findings suggest that the abnormal global and nodal topological properties of the cerebellar functional connectome are associated with CI and could serve as an important biomarker for CI.

## Graphical abstract

Our findings suggest that the abnormal global and nodal topological properties of the cerebellar functional connectome are associated with chronic insomnia and could serve as an important biomarker for chronic insomnia.

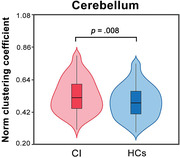

## 1 INTRODUCTION

Chronic insomnia (CI) is characterized by difficulty falling asleep, difficulty maintaining sleep, and early awakening, with these complaints occurring more than three times a week for at least 3 months (Diagnostic and Statistical Manual of Mental Disorders, version 5 [DSM‐V]) (Riemann et al., 2015). It is a neurological syndrome, often accompanied by daytime fatigue, attention deficits, and emotional instability. The incidence of CI is increasing worldwide year by year, and the global prevalence of CI is approximately 30%–35%. The prevalence in individual countries is generally similar (Morin et al., 2015). Previous studies have shown that CI not only impairs patients’ daily functions, but also causes a variety of chronic diseases (such as cardiovascular and psychological disease) that seriously reduce quality of life and may even be life‐threatening (Kyle et al., 2010). In addition, several family and twin genetic studies have confirmed that insomnia is highly hereditary (genetic factor between 42% and 57%) (Barclay et al., 2010; Hublin et al., 2011; Watson et al., 2006). Despite the high incidence and hereditary prevalence of CI, as well as the enormous medical burden and socioeconomic losses (Leger & Bayon, 2010), the exact neurobiological mechanisms of CI remain unknown.

In terms of etiology, CI involves the dysregulation of sleep homeostasis and circadian processes, and sleep–wake cycle abnormalities are fundamental biological processes in CI. Evidence from animal studies indicates that the cerebellum is the input part of the monoamine neurotransmitter regulatory system in the sleep–wake cycle (Canto et al., 2017), and the neurons in the cerebellum contain a variety of neurotransmitter receptors that regulate sleep–wake states. The abnormal activation of these neural receptors can encourage mice to wake up quickly from sleep (Barik & de Beaurepaire, 2005). Previous electrophysiological experiments have found that the destruction of the cerebellar vermis and bilateral cerebellar hemispheres leads to decreased wakefulness phases and increased rapid eye movement, (REM) phases in the physiological sleep cycle of cats (Cunchillos & De Andrés, 1982; de Andres et al., 2011), emphasizing the important role of cerebellar neural activity in the regulation of the sleep–wake cycle. Several neuroimaging techniques have been used to investigate functional changes in the cerebellum in insomnia disorders. Previous functional studies based on positron emission tomography (PET) and functional magnetic resonance imaging (fMRI) have reported insomnia‐related abnormalities in neural function and metabolism of the cerebellum. Previous studies based on PET have found that normal people have significantly reduced cerebellar glucose metabolism levels after 32 h of mandatory sleep deprivation (Wu et al., 1991), and in fatal familial insomnia patients with multiple neurological dysfunctions, cerebellar glucose metabolism decreased (Perani et al., 1993). Whole‐brain amplitude of low‐frequency fluctuation analysis found that regional neuronal activation of the cerebellar bilateral posterior lobe in CI patients was reduced compared with normal people (Li et al., 2016; Zhou et al., 2017). Another study found that the regional concordant activity of the left anterior cerebellar lobe was lower in female CI patients than in normal women (Dai et al., 2014). Whole‐brain functional connectivity analysis based on the seed point showed that the local regions of the cerebellum in CI patients had abnormal functional connection strength with multiple brain regions such as the thalamus, default network, and executive control network (Huang et al., 2017; Pang et al., 2017). Available research presents inconsistencies in the interpretation of cerebellar nerve function in CI patients, and these studies only focus on neuronal activity in the cerebellar region of these patients or changes in the strength of the functional connections between brain–cerebellar neural circuits. The cerebellum performs sleep and cognitive functions by virtue of the synergy of its multiple regions; it has a complex anatomy and extremely rich higher order function (Schmahmann, 1991). Researching the topological properties of the cerebellar connectome and exploring the global/nodal functional coordination of the cerebellum will help develop an understanding of the pathophysiology of the occurrence and development of CI.

Recently, based on a voxel‐wise and node‐wise topological property analysis of the cerebellar functional connectome from 1416 healthy people with resting‐state functional connectivity data, a study discovered that the cerebellar functional connectome is a high‐performance complex network with well‐established topological properties, including small‐world organization, hierarchical institutions, and modular architecture (Chen et al., 2022). This study suggests that analyzing changes in the topological properties of cerebellar functional networks is helpful for understanding the neural mechanisms of the disease. However, few studies have examined whether and how CI alters cerebellar neural network organization from a system‐level perspective of network segregation and integrity. In the present study, we used graph‐based approaches to investigate topological abnormalities of the cerebellar functional connectome in individuals with CI. Among numerous graph theory‐based measures, we exclusively focused on global/nodal functional properties to reveal the possible neurobiological basis of CI at the system level of functional integration/separation, with the aim of providing entirely new ideas for a deeper understanding of the pathological mechanisms of CI.

## 2 MATERIALS AND METHODS

### 2.1 Participants

A total of 203 participants were recruited, including 102 CI patients from the Department of Neurology of Guangdong Second Provincial General Hospital and 101 age‐, gender‐, and education‐level‐matched healthy controls (HCs) recruited from the local community through advertisements from April 2014 to April 2016. All participants were right‐hand dominant, as assessed using the Edinburgh Handedness Inventory (Oldfield, 1971). The present study was approved by the Institutional Review Board of Guangdong Second Provincial General Hospital, and all participants provided written informed consent in accordance with the Declaration of Helsinki.

The diagnostic criteria for CI and HCs was determined by two neurologists with 15–25 years of clinical psychiatric experience each, based on DSM‐V and the International Classification of Sleep Disorders, Third Edition (ICSD‐3), with complaints of difficulty falling asleep, maintaining sleep, or early awakening for at least 3 months and three times per week. Patients with CI were excluded according to the following criteria: (1) insomnia disorder secondary to a severe mental condition (e.g., depression, anxiety, and epilepsy); (2) other sleep disorders; (3) history of significant head trauma or loss of consciousness for >30 min; (4) history of medication‐based treatment for insomnia disorder; (5) history of alcohol abuse, drug abuse, or smoking; (6) abnormal signals on conventional MRI; (7) pregnancy, lactation, or menstruation; and (8) Self‐rating Anxiety Scale (SAS) total score >50 or Self‐rating Depression Scale (SDS) standard score ≥53.

The inclusion criteria for HCs were as follows: (a) good sleep quality and Insomnia Severity Index (ISI) score <7; (b) able to complete MR examination and scale assessments; (c) no brain injury or prior substantial head trauma confirmed by routine T1‐ or T2‐weighted FLAIR; (d) no history of psychiatric or neurological disorders; and (e) not pregnant, breastfeeding, or menstruating (if female).

Each of the CI patients underwent the polysomnographic test and a series of clinical assessments to evaluate their sleep status, sleep behavior, and cognitive mental status, including the Pittsburgh Sleep Quality Index (PSQI) (Buysse et al., 1989), ISI (Bastien et al., 2001), SAS (Zung, 1971), and SDS (Zung, 1965). Each participant underwent a battery of standardized neuropsychological assessments, including Montreal Cognitive Assessment (MoCA) (Hobson, 2015), Rey auditory verbal learning test (RAVLT) (Salgado et al., 2011), and Trail Making Test. Sleep behaviors of each CI participant were evaluated using polysomnographic recordings (UMindSleep, E1) by placing an electrode on the forehead to monitor the electroencephalogram, and the measured sleep parameters included sleep‐onset latency (SOL), sleep efficiency (SE), total sleep time (TST), time in bed, percentage of nonrapid eye movement (NREM) and REM sleep, and wake after sleep onset (WASO). Polysomnographic recordings were conducted the night before MRI scanning. Data for WASO, TIB, and percentage of NREM and REM sleep were excluded because of missing or apparent errors, and finally, TST, SE, and SOL were included in this study.

### 2.2 MRI data acquisition

All participants underwent multimodal MR scanning using a 3.0 Tesla scanner (Ingenia, Philips, The Netherlands) at the Department of Medical Imaging, Guangdong Second Provincial General Hospital.

#### 2.2.1 Resting‐state fMRI data

Resting‐state fMRI (rs‐fMRI) data were acquired using a gradient‐echo planar imaging sequence with the following parameters: repetition time (TR) = 2000 ms; echo time (TE) = 30 ms; flip angle (FA) = 90°; slice thickness = 3.5 mm; gap = 1.0 mm; matrix = 64 × 61; field of view (FOV) = 224 × 224 mm^2^; and 33 transverse planes paralleling the anterior commissure ‐ posterior commissure line (AC‐PC line). The duration of the rs‐fMRI scan was 8 min, and 240 volumes were obtained for each participant. T1‐weighted three‐dimensional magnetization‐prepared rapidly acquired gradient‐echo sequences were obtained with the following parameters: axial slices, 185; TR = 7.9 ms; TE = 3.6 ms; FA = 8°; slice thickness = 1.0 mm (no gap); matrix = 256 × 256; and FOV = 256 × 256 mm^2^. During rs‐fMRI data acquisition, participants were asked to lie quietly with their eyes closed in the scanner and empty their minds of any particular thoughts.

### 2.3 Functional MRI preprocessing

Functional image preprocessing was performed using the GRETNA toolbox (Wang et al., 2015) based on the SPM12 package (http://www.fil.ion.ucl.ac.uk/spm/software/spm12/). First, following the removal of the first five volumes for magnetic saturation, intervolume head motion was corrected via rigid transformation. After converting rotational displacements from degrees to millimeters on the surface of a sphere with a radius of 50 mm (Power et al., 2012), participants with excessive motion were excluded in terms of the criteria of >3 mm translation or >0.5 mm mean frame‐wise displacement. The corrected functional images then underwent band‐pass filtering (0.01–0.08 Hz) and nuisance regression (24‐parameter head motion profiles [Friston et al., 1996], white matter signals, cerebrospinal fluid signals, and global signals) in a single linear model to avoid reintroducing artifacts (Lindquist et al., 2019). White matter and cerebrospinal fluid signals were calculated within subject‐specific masks derived from the tissue segmentation of individual structural images (threshold = 0.9), which were co‐registered to the corresponding mean volume of the corrected functional images. Finally, the functional images were normalized into the standard Montreal Neurological Institute, (MNI) space by applying deformation fields derived from the tissue segmentation of individual structural images and spatially smoothed by a Gaussian kernel (full width at half maximum = 6 mm).

### 2.4 Functional network construction

In this study, we constructed large‐scale individual functional networks based on the correlation coefficients of the time courses between region pairs. A functional brain network comprises a cluster of nodes representing brain regions and edges representing the connectivity between two region pairs. The network nodes were defined using the SUIT atlas (Diedrichsen et al., 2011), which parceled the cerebellum into 28 regions of interest (ROIs). First, the regional mean rs‐fMRI time series was calculated by averaging the time series across all the voxels in that region. The Pearson correlation coefficient of the mean time series between any pair of regions was then calculated to delineate functional connectivity.

### 2.5 Thresholding for functional connections

Before topological analysis of the individual functional networks, we performed a thresholding procedure to exclude spurious connections existing in connectivity matrices constructed based on the correlation coefficient. The thresholds were defined in terms of sparsity (i.e., the real number of edges in the graph divided by the maximum possible number) to ensure that all the derived networks have the same number of edges under the current threshold. The minimum spanning tree algorithm was used in the thresholding procedure to maintain the existence of isolated nodes in the generated networks. Here, we applied a consecutive sparsity threshold ranging from 0.12 to 0.40 with an interval of 0.01, which eliminated the need to define a specific single threshold, simultaneously ensuring that the derived networks remained evaluable for the sparse phenomenon (Achard et al., 2006) and suitable for the subsequent analysis of small‐world properties (Wang et al., 2016; Watts & Strogatz, 1998). The Pearson correlation coefficient matrices were filtered by the consecutive sparsity threshold, resulting in the final weighted and undirected functional networks.

### 2.6 Functional network properties

For each network, we computed six properties, consisting of four global and two nodal properties. The four global properties are the clustering coefficient (i.e., the mean value of the local interconnectivity or cliques among the neighbors of all nodes in a network), characteristic path length (i.e., the mean value for the smallest sum of distances among all possible paths from one node to another in a network), normalized clustering coefficient, and normalized characteristic path length. The two nodal properties were nodal degree (i.e., the number of edges between the given node and the rest of the nodes) and nodal betweenness centrality (i.e., the influence of the given node on the information flow among all the other nodes). The normalized global properties mentioned above were used to determine whether the functional networks were nonrandomly organized, and they were generated by normalizing the real value by the corresponding mean value of 1000 matched random networks. A topological rewiring algorithm (Maslov & Sneppen, 2002) was employed in the normalization to maintain the same degree distribution between random networks and real networks. Notably, we calculated the area under the curve (i.e., the integral over the sparsity range) for each global and nodal property to enable all properties to be independent of the thresholds and to simplify subsequent statistical analyses.

### 2.7 Statistical analysis

#### 2.7.1 Clinical data

Comparisons of demographic factors (age, sex, and years of education) and clinical assessment data between CIs and HCs were performed using the chi‐squared test; two‐sample *t*‐tests and Wilcoxon rank‐sum tests were used for independent data. Statistical analyses were performed using SPSS 21.0. All quoted results were two‐tailed, and *p* < .001 was considered statistically significant.

#### 2.7.2 Between‐group differences

Between‐group differences in the network measures were inferred using nonparametric permutation tests. An empirical distribution of the *T*‐statistic was then obtained by randomly reallocating all values into two groups and recomputing the *T*‐statistic between the two randomized groups (10,000 permutations). The 95th percentile points of the empirical distribution were used as critical values in a two‐tailed test to determine whether the observed group differences could occur by chance. The *p*‐value of the real observation was measured as the proportion of permutations that showed higher absolute statistics than the real *T*‐statistic among the 10,000 permutations. To correct for multiple comparisons of network properties, the false discovery rate (FDR) procedure was used at the level of *q* < .05 (global properties: 4 properties × 1 ROI; nodal properties: 2 properties × 28 ROIs).

#### 2.7.3 Cerebellum–behavior relationships

For each network property that showed significant alterations in the patient group, partial correlation analysis was used to assess the relationships between the altered network metrics and clinical variables, sleep behavior, and cognitive performance in the CI group after controlling for age, sex, mean framewise displacement of head motion, and years of education. Multiple comparisons were adjusted using FDR correction.

## 3 RESULTS

### 3.1 Demographic and clinical assessments

There were no significant differences in sex, age, or years of education between the CI and HC groups. There were statistically significant differences in RAVLT delayed recall, MoCA total score, MoCA visual–spatial executive, MoCA attention, and MoCA delayed recall between the CIs and HCs (Table 1).

**TABLE 1:** Demographics and clinical assessments of participants

	CIs (*n* = 102)	HCs (*n* = 101)	*p*‐value
Demographics			
Gender (M/F)	24/78	32/69	.194^a^
Age (years)	45.5 (23)	45 (21)	.913^b^
Education (years)	15 (12–16)	16 (12–16)	.067^b^
Clinical assessments			
Trail Making Test A	49 (29)	50 (20)	.883^b^
Trail Making Test B	126.5 (76)	113 (72.25)	.056^b^
RAVLT immediate recall	42 (14)	44 ± 9.534	.011^b^
RAVLT delayed recall	3 (2)	4 (2.25)	**<.001** **^b^**
MoCA total score	24 (5)	27 (4)	**<.001** **^b^**
MoCA visual–executive function	4 (2)	5 (1)	**<.001** **^b^**
MoCA naming	3 (0)	3 (0)	.026^b^
MoCA attention	6 (2)	6 (0.25)	**<.001** **^b^**
MoCA language	2 (1)	3 (1)	.019^b^
MoCA abstraction	2 (1)	2 (0)	.023^b^
MoCA delayed recall	3 (2)	4 (1)	**<.001** **^b^**
MoCA orientation	6 (0)	6 (0)	>.999^b^
Digit Symbol Test (DST)	46.471 ± 16.416	55 (17.25)	.043^b^
Digit Span Test (DST)	13 (3)	14 (3)	.020^b^
PSG sleep onset latency (min)	30 (30)	–	–
PSG sleep efficiency	0.71 (0.25)	–	–
PSG total sleep time (h)	5 (2)	–	–
PSQI	13 (5)	–	–
ISI	16 (7)	–	–

*Note*: Data are represented as mean ± standard deviation or median (interquartile range) for continuous variables, depending on whether the variables follow a normal distribution (Lilliefors test). For the neurophysiological tests, the Bonferroni method was used to correct for multiple comparisons, and significant between‐group differences are shown in bold font.

Abbreviations: CIs, chronic insomnia patients; HCs, healthy controls; ISI, Insomnia Severity Index; MoCA, Montreal Cognitive Assessment; PSG, polysomnography; PSQI, Pittsburgh Sleep Quality Index; RAVLT, Rey Auditory Verbal Learning Test.

^a^ The *p*‐value was obtained with a chi‐squared test.

^b^ *p*‐values were obtained using two‐sample *t*‐tests or Wilcoxon rank sum tests.

### 3.2 Global properties of functional cerebellar networks

**FIGURE 1:**
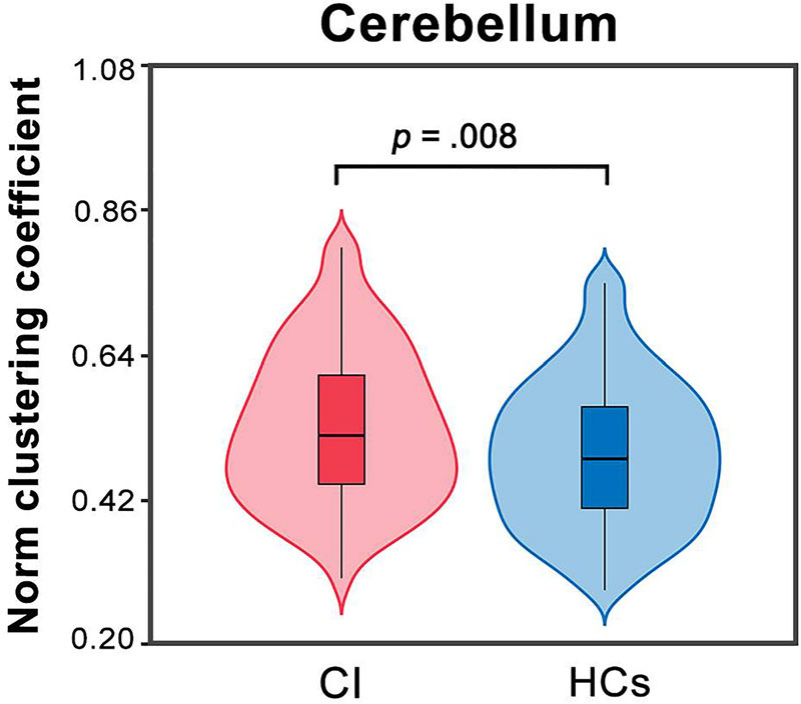
Graph‐based network analysis reveals significant differences between the two groups. CI patients showed increased normalized clustering coefficient in functional cerebellar connectome (*p* = .008) compared with HCs. CI, chronic insomnia; HCs, healthy controls.

We constructed individual functional cerebellar networks by calculating interregional functional connectivity among the 28 ROIs. After applying a significance‐based threshold, the number of surviving connections and their associated weights showed no significant between‐group differences (all *p* > .05). Nevertheless, statistical comparisons revealed significant differences in the quantitative network measures between the two groups. When normalized by random networks, the normalized clustering coefficient in the CI patients was higher (*p* = .008) than that in the HCs (Figure 1).

### 3.3 Nodal properties of functional cerebellar networks

**FIGURE 2:**
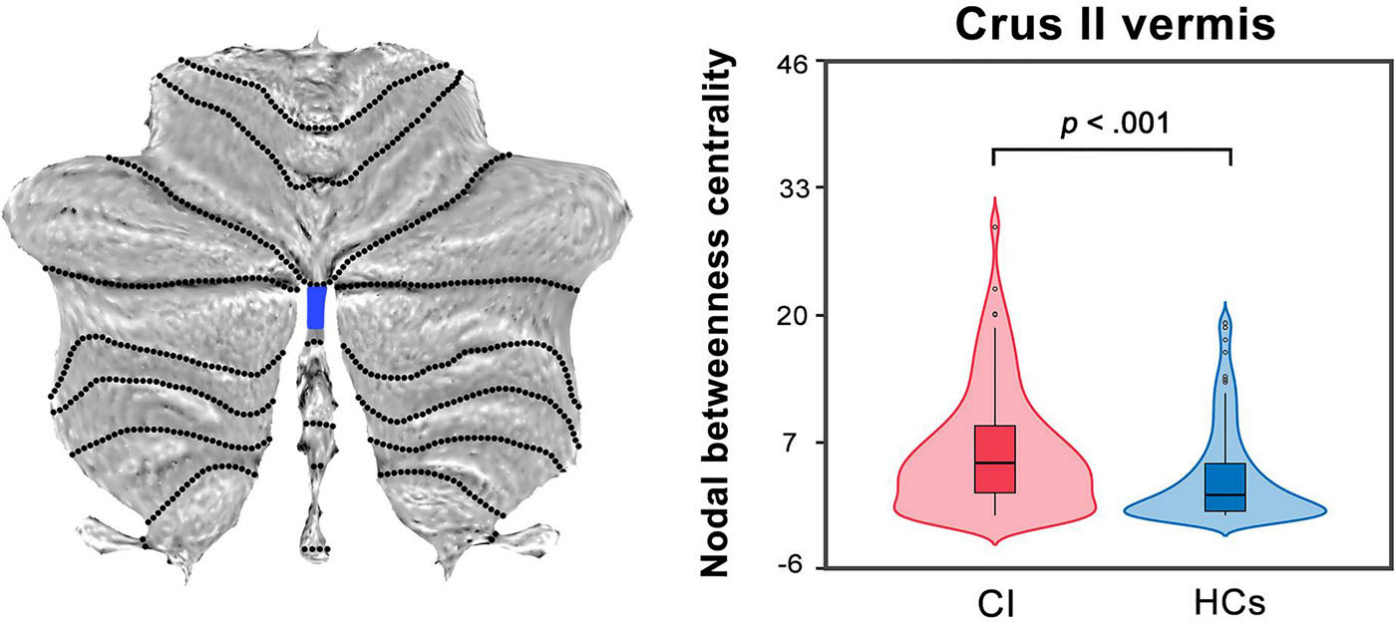
Compared with HCs, CI patients showed a significantly increased nodal betweenness centrality (*p* < .001) in cerebellar Crus II vermis regions. CI, chronic insomnia; HCs, healthy controls.

We constructed functional cerebellar networks by calculating the nodal properties of the nodal degree and betweenness centrality. Compared with HCs, CI patients showed abnormal topology characterized by high nodal betweenness centrality (*p* < .001) in the cerebellar Crus II vermis regions (Figure 2).

### 3.4 Relationship between altered global/nodal properties and clinical assessments

There was no significant correlation between altered global/nodal properties (normalized clustering coefficient, betweenness centrality of cerebellar Crus II vermis) and clinical assessments (RAVLT delay recall, MoCA total score, MoCA visual exe, MoCA attention, MoCA delay recall, insomnia duration, polysomnography [PSG] sleep onset latency, PSG sleep efficiency, PSG total sleep time, PSQI, ISI) in patients with CI (*p* > .05, false discovery rate corrected). Specifically, the normalized clustering coefficient was negatively correlated with total sleep time (*p* = .013, uncorrected) and positively correlated with PSQI (*p* = .017, uncorrected) (Table 2) and ISI (*p* = .015, uncorrected) (Table 2).

**TABLE 2:** Relationships between altered global/nodal properties and clinical assessments

	Norm clustering coefficient	Nodal betweenness centrality of Crus II vermis
RAVLT delay recall	−.113 (.258)	.047 (.638)
MoCA total score	.001 (.995)	.027 (.788)
MoCA visual exe	.026 (.792)	−.002 (.988)
MoCA attention	−.032 (.747)	−.009 (.927)
MoCA delay recall	.018 (.858)	.132 (.185)
PSG sleep onset latency	.078 (.436)	.059 (.558)
PSG sleep efficiency	−.137 (.169)	.065 (.514)
PSG total sleep time	−.245 (.013)	.008 (.938)
PSQI	.236 (.017)	.048 (.634)
ISI	.240 (.015)	.022 (.827)

*Note*: Data are represented as correlation coefficient (*p*‐value).

Abbreviations: ISI, Insomnia Severity Index; MoCA, Montreal Cognitive Assessment; PSG, polysomnography; PSQI, Pittsburgh Sleep Quality Index; RAVLT, Rey Auditory Verbal Learning Test.

## 4 DISCUSSION

This study used the topological properties of the cerebellar functional connectome approach to identify global and nodal property changes in the cerebellar functional connectome of CI patients and HCs and their correlations with indicators of clinical sleep and cognition. This study reports two main findings: (a) Global properties: the normalized clustering coefficient of the cerebellar functional connectome was higher in CI patients than in HCs; (b) Nodal properties: betweenness centrality increased in the cerebellar Crus II vermis region of CI patients relative to HCs. Although changes in topological properties of CI are not correlated with clinical sleep and cognition after multiple comparison correction, our results reveal the possible neurobiological basis of CI from the systemic level of cerebellar functional networks, providing new ideas for understanding the pathological mechanism of CI, in order to guide treatment and predict insomnia.

The human brain is a highly nonlinear system of complex networks that enables complex functions to be performed through the integration of information. This study focuses on four global properties of the cerebellar connectome: clustering coefficient, characteristic path length, normalized clustering coefficient, and normalized characteristic path length. These coefficients represent the functional separation and integration of functional networks in the cerebellum, and their alterations are closely related to cognitive function and brain disease (Wang et al., 2021). At the global level, CI patients showed higher normalized clustering coefficients, implying an increased degree of specialization in local information processing in the cerebellar functional connectivity group of CI patients, which led to an increased ability to exchange information in the cerebellar functional connectivity group of CI patients. We found that CI patients present with increased local information connectivity in the cerebellar functional network consistent with previous studies reporting increased functional connectivity in the intrinsic connectivity network of CI patients (Chen et al., 2014; Leerssen et al., 2019). A higher number of connections between local nodes may lead to more efficient pathways and therefore more efficient local information dissemination and exchange, impling an overactive functional separation of the cerebellum, which is consistent with the insomnia hyperarousal model (Riemann et al., 2010). Our findings differ from those of previous studies on abnormal functional alterations in CI that have focused only on functional alterations in local regions of the cerebellum; we use the cerebellum as a whole to study altered cerebellar neural circuits and the separation and integration of functions between different subregions within the cerebellum in CI. We suggest for the first time a potential mechanism for the cerebellar functional connectome in CI, indicating that intrinsic functional properties of the cerebellum (nonmotor functions) also play a role in sleep–wake regulation in CI. In addition, we did not find differences in characteristic path length, normalized characteristic path length, and clustering coefficients between the two groups, which may be due to insufficient statistical power or more complex disorders in the cerebellar functional connectome of CI patients compared to the cerebrum. Further studies will expand the sample.

Interestingly, we found a correlation between normalized clustering coefficients and sleep alterations (total sleep time, PQSI, and ISI scores). Reduced sleep duration is a common sleep alteration in CI, and one study found that subjects experiencing prolonged sleep duration reduction developed cognitive performance deficits (Dongen et al., 2003). In addition, the PQSI is an assessment of sleep quality (Ong et al., 2011) and the ISI is an assessment of insomnia severity (Morin et al., 2011), both of which are commonly used tools to assess sleep function. Therefore, our findings suggest that altered topography of the cerebellar functional network may be the cause of altered sleep rhythms and sleep dysfunction in patients with CI. After correction for multiple comparisons, this result is not statistically significant, possibly due to the influence of multiple confounding factors, and future research is needed with long‐term, longitudinal, large samples.

In terms of nodal properties, we found that CI patients had increased betweenness centrality in the cerebellar Crus II vermis region compared to HCs, but no correlation was found between it and clinical sleep and neurocognitive scales. Betweenness centrality is the proportion of all shortest paths through a given node in the network . Thus, bridging nodes that connect different parts of the network produce high betweenness centrality. Increased betweenness centrality of the cerebellar Crus II vermis in CI patients represents increased and enhanced functional connectivity of this node with other nodes in the network, so this node is more prominent and important in CI patients than in HCs and its ability to transmit information is increased. A previous meta‐study of cerebellar functional topography found that the cerebellar Crus II vermis showed functional activation in emotional processing tasks and language tasks, indicating that it is associated with emotional regulation and language processing (Stoodley & Schmahmann, 2009). Another study showed that in a population of university students with English as a second language, the severity of their insomnia closely correlated with their language anxiety (Shen & Gellis, 2021). The above results implying that the enhanced function and increased information transmission capacity of the cerebellar Crus II vermis region may be a potential mechanism help to compensate for language processing disorders in people with CI. In addition, the cerebellar vermis is primarily involved in processing emotional and fear‐related responses, including anxiety (Reiman, 1997), grief (Gündel et al., 2003), and pain (Dimitrova et al., 2004). Previous studies have indicated that anxiety symptoms and difficulties in emotion regulation contribute to severe insomnia symptoms (Palagini et al., 2017), and many etiological theories suggest that enhanced emotional responses contribute to the maintenance of insomnia symptoms (Vanek et al., 2020). Our results suggest that abnormal cerebellar Crus II vermis region function may be a potential mechanism for the long‐term maintenance of sleep disturbances in CI. Recent studies have indicated that the cerebellar Crus II vermis, as a separate neurofunctional entity, maps largely to the default mode component and associative cortex (Buckner et al., 2011; Schmahmann et al., 2019) and is involved in a variety of cognitive functions such as social processing and working memory (Guell et al., 2018; Keren‐Happuch et al., 2014). A study based on graph theory analysis proposed that the Crus II region is a key node or topological hub in the functional cerebellar connectome with high centrality (Chen et al., 2022). In our study, the increased betweenness centrality of the Crus II vermis region suggests that in CI patients, synchronization with functional brain processes may be improved by increasing the mapping of area Crus II in the cerebral cortex, thereby compensating cognitive impairment. The enhanced betweenness centrality of the cerebellar Crus II vermis suggests that there may be some compensatory mechanism to mitigate the impairment of cerebellar cognitive function and the sleep–wake cycle in CI.

Our study has several limitations. First, we need further studies to determine whether these altered cerebellar functional connectome topological properties in CI patients can be replicated and whether they change with disease progression and treatment. Second, this study only explored topological alterations in the cerebellar connectome between CI patients and HC controls and did not develop topological properties of the cerebral connectome; therefore, the specific cerebellar–cerebral mapping of CI patients is unclear. Finally, the bias correlation analysis in this study showed no correlation, and the relationship between topological abnormalities in the cerebellar functional connectome and clinical phenotypes was unclear, possibly because the length of illness and heterogeneity of the main clinical phenotypes in CI patients influenced the changes in topological properties of the cerebellar functional connectome. Therefore, in future studies, we will use a more homogeneous sample to refine our conclusions. This also suggests that future studies can further group CI patients according to the duration of the disease and clinical features, thus better exploring the pathophysiological mechanisms of different subgroups of CI patients and contributing to individualized clinical treatment.

## 5 CONCLUSION

To our knowledge, this is the first study to examine the abnormal topology of the functional connectome of the inner cerebellum in patients with CI. We found that both patients with CI symptoms and HCs without insomnia symptoms exhibited small‐world characteristics. Based on a measure of global topological properties, patients with CI had higher standardized clustering coefficients than HCs. Nodal properties of the cerebellar Crus II vermis region had increased betweenness centrality relative to HCs. Thus, our findings present new evidence that global and nodal property disruption in the topological organization of the cerebellar functional connectome may be a cause or consequence of insomnia.

## AUTHOR CONTRIBUTIONS

Siqi Lin and Xi Ye conceived, designed, and performed the experiments; contributed reagents/materials/analysis tools; and wrote and revised the original manuscript. Yuping Yang and Jingyi Yang analyzed the data and constructed the figures. Xinzhi Wang and Jingyi Yang contributed reagents/materials/analysis tools. Guang Xu performed the experiments. Xiaofen Ma conceived, designed, and performed the experiments.

### PEER REVIEW

The peer review history for this article is available at https://publons.com/publon/10.1002/brb3.3103

## Data Availability

n/a.
